# Manual (a)symmetries in grasp posture planning: a short review

**DOI:** 10.3389/fpsyg.2014.01480

**Published:** 2014-12-15

**Authors:** Christian Seegelke, Charmayne Mary Lee Hughes, Thomas Schack

**Affiliations:** ^1^Neurocognition and Action Research Group, Faculty of Psychology and Sport Sciences, Bielefeld UniversityBielefeld, Germany; ^2^Center of Excellence Cognitive Interaction Technology, Bielefeld UniversityBielefeld, Germany; ^3^Robotics Research Centre, School of Mechanical and Aerospace Engineering, Nanyang Technological UniversitySingapore, Singapore; ^4^Research Institute for Cognition and Robotics (CoR-Lab), Bielefeld UniversityBielefeld, Germany

**Keywords:** manual asymmetries, grasping, motor planning, end-state comfort, bimanual coordination

## Abstract

Many activities of daily living require that we physically interact with one or more objects. Object manipulation provides an intriguing domain in which the presence and extent of manual asymmetries can be studied on a motor planning and a motor execution level. In this literature review we present a state of the art for manual asymmetries at the level of motor planning during object manipulation. First, we introduce pioneering work on grasp posture planning. We then sketch the studies investigating the impact of future task demands during unimanual and bimanual object manipulation tasks in healthy adult populations. In sum, in contrast to motor execution, there is little evidence for hand-based performance differences in grasp posture planning. We discuss potential reasons for the lack of manual asymmetries in motor planning and outline potential avenues of future research.

## INTRODUCTION

The study of differences in the performance capabilities of the two hands, commonly referred to as manual asymmetries, has long been a topic of intense study among researchers from fields such as psychology, neurophysiology, and motor control (see Goble and Brown, 2008, for a review). It is commonly accepted that humans prefer to use one hand over the other when performing manual everyday tasks (e.g., writing or grasping an object), with the majority of people (about 90% of the population) exhibiting a preference to use the right hand over the left (Coren and Porac, 1977). Considering the performance of the two hands, it has been reported that task performance with the dominant hand is often superior compared to the non-dominant hand. For example, the dominant arm of right-handed individuals can produce greater forces than the non-dominant hand (e.g., Petersen et al., 1989; Armstrong and Oldham, 1999), is faster and more consistent during repetitive finger tapping (Peters, 1976; Todor and Kyprie, 1980; Todor et al., 1982) and is more accurate during reaching and rapid aiming movements (Annett et al., 1979; Roy and Elliott, 1989; Carson et al., 1993).

Investigations into manual asymmetries are not limited to the level of motor execution but have also been extended to the motor planning level. One intriguing domain in which motor planning can be studied is object manipulation (see Rosenbaum et al., 2012, 2013, for reviews). As the very same object can be grasped differently depending on whether one intends to use that object or to pass it to another person to use, differences in the way an object is grasped depending on different future task demands or action goals can be ascribed to differences in the respective action plans. In addition, object manipulation provides the opportunity to study motor planning of different orders (Rosenbaum et al., 2012). Whereas first-order planning reflects adjustments of grasp postures to immediate task demands (e.g., object orientation, shape, and size), second-order planning reflects adjustments that not only consider immediately available perceptual information but also incorporate demands of the next task to be performed.

In this article, we review current research on second-order motor planning during object manipulation tasks (i.e., grasping an object with *one* subsequent displacement), with a focus on the impact that future task demands elicit on the presence of manual asymmetries.

## PIONEERING WORK

The foundation of second-order motor planning in the context of object manipulation was inspired by a natural observation David A. Rosenbaum made in a restaurant where he observed a waiter pouring water into drinking glasses. The glasses stood inverted on the table. To fill each glass with water, the waiter initially grasped it with a (presumably uncomfortable) thumb-down grip, turned it by 180∘ to set it down with a (comfortable) thumb-up grip. Rosenbaum et al. (1990) transferred this observation to the laboratory. The setup used in this study – which has become known as the ‘bar-transport task’ – consisted of a wooden bar which was horizontally arranged on a cradle such that participants could grasp it using either an overhand grasp or an underhand grasp. Participants grasped the bar and rotated it 90∘ to place either its left or right end into a target disk located to the left or right side. The authors found that, regardless of target location, the selection of initial grasp posture (i.e., underhand or overhand grasp) depended on the required end orientation of the bar. Specifically, participants adopted an initial overhand grasp posture when using the dominant right hand to place the right end of the bar into the target disk. Conversely, when the left end of the bar was to be placed into the target disk, participants initially grasped the bar with an underhand grasp. Thus, participants always selected an initial grasp that afforded a comfortable thumb-up posture at the end of the movement. Termed the end-state comfort effect, this phenomenon indicates that participants represent future posture states and plan their initial grasps in anticipation of these future postures prior to movement execution. The end-state comfort effect provides a nice tool to study motor planning processes and it has been applied in a variety of different tasks (e.g., Cohen and Rosenbaum, 2004; Herbort and Butz, 2010, 2012; Hughes et al., 2012c). Consequently, the end-state comfort effect is also an instrument to examine whether manual asymmetries are evident on a motor planning level during unimanual and bimanual object manipulation tasks. Work on these topics will form the focus of the following two sections.

## UNIMANUAL TASKS

**FIGURE 1:**
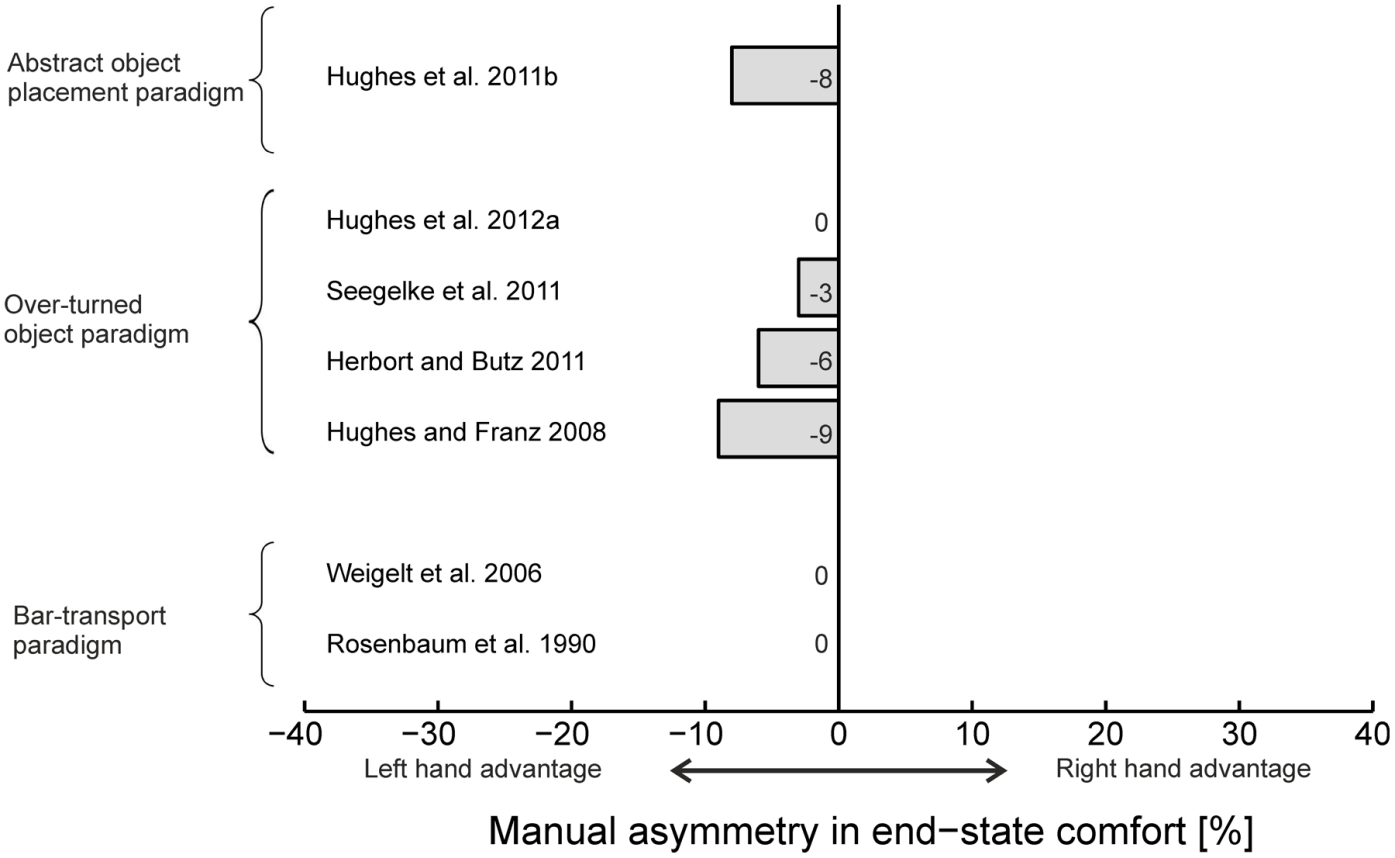
**Assessment of manual asymmetries in end-state comfort in unimanual tasks.** Negative values indicate higher end-state comfort for the left hand, positive values indicate higher end-state comfort for the right hand. For Herbort and Butz (2011) values are based on participants who exclusively used thumb-up and thumb-down grasps. Results from Rosenbaum et al. (1993) are not shown as no exact values are presented in this paper.

In the Rosenbaum et al. (1990) study participants were initially not told which hand to use when performing the task. The authors reported that out of the 12 participants, six participants used only their right hand, one participant used only the left hand, and the remaining five participants switched hands between trials. Nevertheless, independent of hand choice, participants always selected initial grasp posture that were in accordance with the end-state comfort effect. Thus, left hand performance mirrored right-hand performance. Similar results using the bar-transport task were obtained by Weigelt et al. (2006; see **Figure 1**).

In a later study, Rosenbaum et al. (1993) employed a task that allowed for a more fine-grained measure of motor planning performance. The experimental setup consisted of a handle connected to a disk. A small cardboard tab was attached to the disk such that rotating the handle caused the disk and the tab to turn. The tab covered one of eight target position which were arranged around the disk (separated by 45∘). In this task, participants grasped and turned the handle such that the tab would cover a designated target, and each possible combination of start and end position were tested. Confirming the results of the original bar-transport task, initial grasp postures depended on the final target position and were selected to afford a comfortable end posture. Again, there was no evidence for manual asymmetries in motor planning as left-hand performance mirrored that of the right hand.

Subsequent research on grasp posture planning during unimanual object manipulation tasks has also reported equal performance between hands (Hughes and Franz, 2008; Herbort and Butz, 2011; Hughes et al., 2011b, 2012a; Seegelke et al., 2011; see **Figure 1**). For example, in the first study specifically conducted to investigate the presence and extent of manual asymmetries on motor planning (Seegelke et al., 2011), participants grasped a vertically oriented cylinder with the dominant right hand, or the non-dominant left hand, and placed it to a target located to the left or right side of the object’s start position. Thus, in contrast to the original bar-transport task which necessitated always 90∘ object rotation, in this paradigm the object was to be placed vertically to the targets such that it required either no rotation or 180∘ rotation, depending on condition. Based on the literature regarding manual asymmetries in motor execution (cf. Elliott and Chua, 1996), it was hypothesized that the dominant right hand should exhibit a greater preference for comfortable end postures than the non-dominant left hand. In that study it was observed that initial grasp selection was strongly influenced by target location and the required object end-orientation. However, the hypothesis regarding manual asymmetries was not confirmed. Regardless of target location or the hand used to move the object participants almost always used a thumb-up grasp posture during trials in which the object required no rotation. During trials that required 180∘ object rotation trials, it was found that end-state comfort satisfaction was significantly more pronounced for the contralateral target location for both the dominant and the non-dominant hand. Analogous to the well-established notion that spatial precision demands affect the presence and extent of manual asymmetries during motor execution (e.g., Bryden and Roy, 1999; Bryden et al., 2007; van Doorn, 2008), it was reasoned that the absence of manual asymmetries might be rooted in the relatively low precision requirements of the task.

However, this explanation was soon rendered unlikely by a subsequent study in which the precision demands at the start and the end of the movement were manipulated (Hughes et al., 2012a). In this task, participants grasped a vertically arranged cylinder located in a start disk with the left or right hand and placed it vertically to a target disk with either no or 180∘ object rotation. The diameter of the start and target disks were manipulated so that the precision requirements were either identical (low initial and final precision, high initial and final precision) or different (low initial high final precision, high initial low final precision). The general finding was that half of the participants (precision-sensitive group) adjusted their initial grasps depending on the precision requirements of the task (i.e., they adopted comfortable postures at the position where the precision demands were high), whereas the other half (end-state comfort consistent group) planned their movements such that they would satisfy end-state comfort regardless of precision demands. However, and of greater importance for the purpose of this review, there were no differences in grasp choice between the dominant right and the non-dominant left hand for either subset of participants (overall end-state comfort satisfaction: precision-sensitive: left hand 64%, right hand 62%; end-state comfort consistent group: left hand 97%, right hand 99%). Taken together, the results from this study provide evidence that precision demands do not affect manual asymmetries on a motor planning level.

In another study (Herbort and Butz, 2011), participants grasped an upright or inverted cup with either the dominant right or the non-dominant left hand and rotated it by 180∘ before placing it on the target circle. The authors found that initial cup orientation significantly affected grasp choice. Inverted cups were grasped more frequently with an initial thumb-down grasp whereas upright cups were grasped more often with an initial thumb-up grasp. However, the hand used for object manipulation did not affect grasp choice for either the upright or inverted cup orientation. It was argued that the inability to detect manual asymmetries might be due to the low complexity level of their task, or that participants had to interact with a common everyday object (i.e., a drinking cup), for which stereotypic (habitual) solutions already exist. The authors postulated that more complex actions – for example bimanual actions – might provide a more suitable situation in which potential hand-based differences in motor planning may be observed.

## BIMANUAL TASKS

A number of researchers have been interested in whether the end-state comfort effect would extend to movements made with the two hands (Fischman et al., 2003; Weigelt et al., 2006; Hughes and Franz, 2008; van der Wel and Rosenbaum, 2010; Hughes et al., 2011a,b). Bimanual movements provide an interesting scenario in which to examine grasp posture planning, as the sensitivity toward end-state comfort often competes with the strong tendency for the two hands to grasp objects with identical postures (bimanual spatial coupling).

The first report of manual asymmetries in bimanual movements on a motor planning level came from the work of Janssen et al. (2009). In this study, participants simultaneously grasped two CD casings (one with each hand) from two lower boxes and place them into two upper boxes. The authors manipulated the start and end orientation of each CD (horizontal or vertical), the start and end orientation congruency (congruent: both CDs horizontal or vertical; incongruent: one CD horizontal, one CD vertical) and the required object rotation (0∘, 90∘ supination, 90∘ pronation, 180∘). The experiment was designed such that one CD always required 180∘ rotation while the other required 0∘, 90∘ supination or 90∘ pronation. Janssen et al. (2009) found that the tendency of right-handed individuals to avoid uncomfortable end-postures was higher and more variable for the right hand (82.0%, SD = 20.2%) than for the left hand (49.8%, SD = 9.8%). However, the sensitivity toward end-state comfort was strongly influenced by object end-orientation, such that the tendency to avoid uncomfortable end-postures was higher when the CD was to be placed in a vertical (80.8%, SD = 11.3%), than in a horizontal end-orientation (61.9%, SD = 15.7%). Janssen et al. (2009) argued that the presence of manual asymmetries observed in their study arose from the increased complexity of the CD placement task compared to the bar transport paradigms used in previous studies that either did not observe or failed to report the presence of manual asymmetries (Fischman et al., 2003; Weigelt et al., 2006). This increased complexity resulted in a breakdown of overall anticipatory planning performance with participants prioritizing end-state comfort planning for the dominant right hand.

The authors posited that the observed manual asymmetries in end-state comfort compliance occurred because of differences in hemispheric specializations with respect to motor planning, and tested this *left-hemisphere dominance for motor planning hypothesis* by asking left-hand dominant individuals to perform the CD placing task (Janssen et al., 2011). As in their previous study (Janssen et al., 2009) they found that end-state comfort was more pronounced for the right hand, compared to the left hand, especially during movements to horizontal end-orientations. The similarity between both handedness groups was congruent with the expectations of the left-hemisphere dominance for motor planning hypothesis, hereby bolstering the claim that motor planning is a specialized function of the left hemisphere (Kim et al., 1993; Haaland et al., 2000; Frey, 2008).

Motivated in part by the results of Janssen et al. (2009, 2011), Hughes et al. (2011a) explored hemispheric differences in motor planning and execution in left- and right-handed individuals in a grasping and placing task in which participants grasped two objects from a table and placed them on a board to one of four end-orientations (0∘, 90∘ internal rotation, 180∘rotation, 90∘external rotation). Manual asymmetries in motor execution were observed, with shorter object transport times observed for the left hand, regardless of handedness. However, contrary to the left hemisphere dominance motion planning hypothesis, end-state comfort sensitivity was similar for both the non-dominant and dominant hand, regardless of whether the individuals were left- or right-handed.

Hughes et al. (2011a) suggested that the discrepancy in results between Janssen et al. (2009, 2011) and their study arose due to differences in task paradigm. In the grasping and placing paradigm we employed, participants were required to place the objects on a fitting board, whereas in the studies of Janssen et al. (2009, 2011) participants placed a CD casing into a box. Thus, it could be argued that the CD placing task required a higher level of precision at the end of the movement than placing an object on a fitting board, and that the planning of initial grasp postures is influenced by the precision demands of the task. The authors argued that this hypothesis was unlikely to account for differences across paradigms, as participants in Hughes et al. (2011a) were very accurate when placing the object on the fitting board, and other studies that also employed high precision tasks (e.g., Weigelt et al., 2006) reported that participants almost always complied with end-state comfort, regardless of hand. Based on these pieces of evidence Hughes et al. (2011a) mentioned the possibility that the manual asymmetries in motor planning were specific to the CD placement task and paradigm.

**FIGURE 2:**
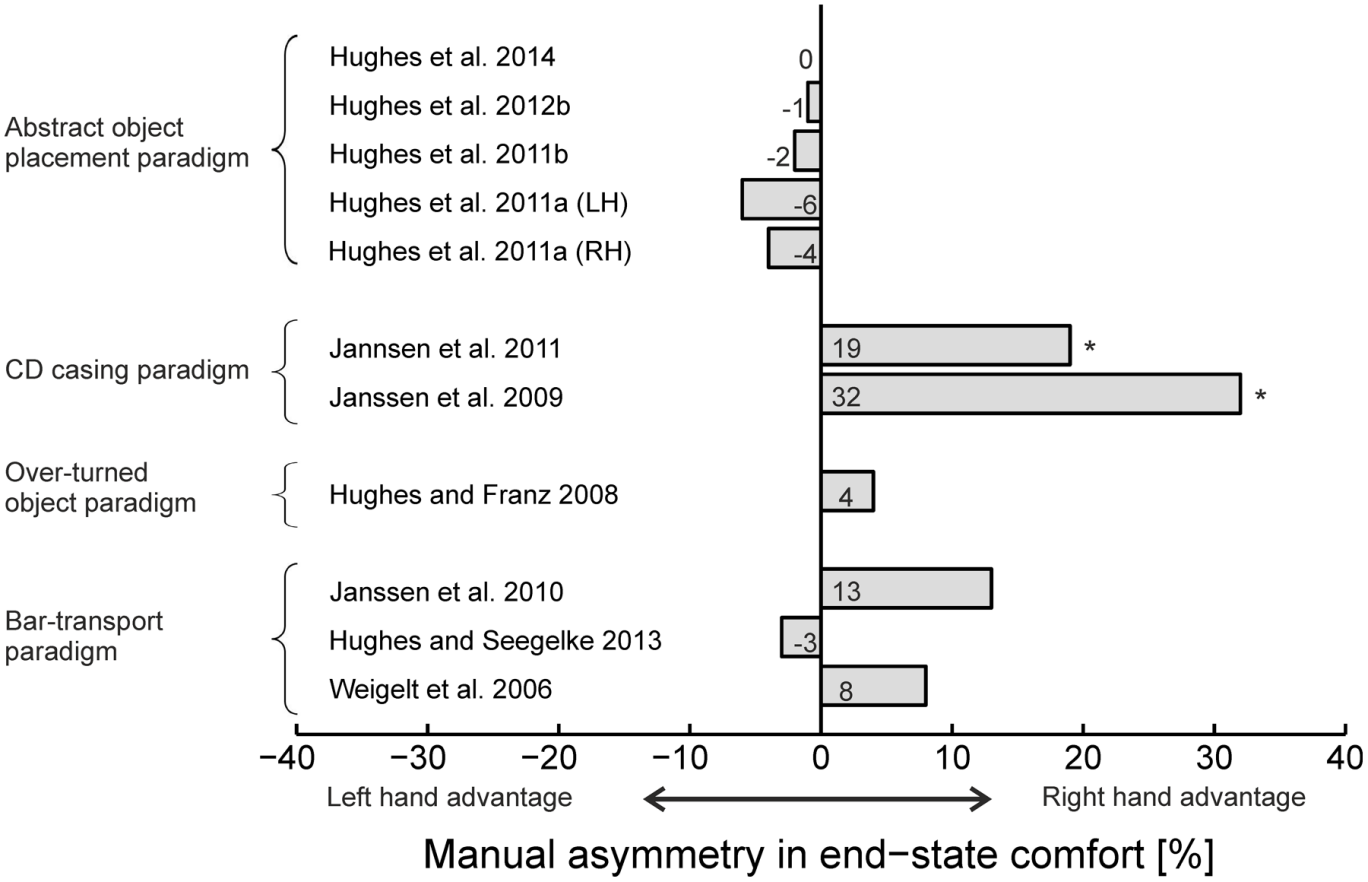
**Assessment of manual asymmetries in end-state comfort in bimanual tasks.** Negative values indicate higher end-state comfort for the left hand, positive values indicate higher end-state comfort for the right hand. Asterisks indicate significant differences between the hands. LH = left handers, RH = right handers. Results from Fischman et al. (2003) and van der Wel and Rosenbaum (2010) are not shown as no exact values are presented in these papers.

While this latter issue is still open for debate, considering all literature on bimanual end-state comfort available at the present time, there is little evidence to support the existence of manual asymmetries at the motor planning level. Besides the higher end-state comfort values for the right hand in the CD placement task (Janssen et al., 2009, 2011), similar end-state comfort compliance for the two hands have been reported in the following bimanual paradigms: bar transport paradigm (Fischman et al., 2003; Weigelt et al., 2006; Hughes and Seegelke, 2013), plunger transport paradigm (van der Wel and Rosenbaum, 2010), over-turned object paradigm (Hughes and Franz, 2008), bar-and-spoon rotation paradigm (Janssen et al., 2010), and abstract object placement paradigm (Hughes et al., 2011a,b, 2012b, 2014; see **Figure 2**).

## CONCLUSION

In this short review we found little evidence for hand-based performance differences in grasp posture planning during second-order object manipulation tasks in healthy adults. These observations are in contrast to the routinely reported presence of manual asymmetries on the level of motor execution. Motor planning and motor execution constitute different (though temporally overlapping) stages of human motor behavior (see Glover, 2004, for a review), and there exists considerable evidence from behavioral (e.g., Woodworth, 1899; Keele and Posner, 1968; Meyer et al., 1988) and neurophysiological studies demonstrating a functional distinction between these two stages (e.g., Grol et al., 2007; Glover et al., 2012; Begliomini et al., 2014). Given this differentiation, it seems reasonable to assume that task constraints known to influence manual asymmetries during motor execution [e.g., precision demands of the task (Bryden and Roy, 1999; Bryden et al., 2007), task complexity (cf. Bryden, 2000)] may not equally affect performance differences between the hands on the level of motor planning.

It has been argued that motor planning of complex actions (i.e., actions beyond simple reaching and pointing) involves decisions about the shape of the trajectory in an effector-independent manner (i.e., abstract kinematics; see Wong et al., 2014). The existence of such abstract goal representations has received support from numerous behavioral and neurophysiological studies (e.g., Keele, 1981; Wright, 1990; Castiello and Stelmach, 1993; Rijntjes et al., 1999; Wing, 2000; van der Wel et al., 2007; Albert and Ivry, 2009; Fu et al., 2010; Swinnen et al., 2010; Sartori et al., 2013) and is encompassed by the notion of *motor equivalence* – the capability of the motor system to achieve the same action goal by different means (Lashley, 1930, 1933; see also Bernstein, 1941). Consequently, the equal performance capabilities of the two hands suggest that decisions about which grasp posture to adopt are done without considering the effector used to execute that action, and reflect hand-independent motor planning processes at high levels of the motor hierarchy that are engraved through lifelong practice.

Alternatively, it is possible that the insensitivity of the measures may have masked manual asymmetries in grasp planning. The bulk of the studies conducted so far examined grasp posture planning using a binary grasp choice (i.e., participants could adopt only one of two grasps; e.g., underhand vs. overhand; thumb-down vs. thumb-up). As such, it is possible that manual asymmetries in grasp posture planning may be detected (if indeed they do exist) by employing continuous instead of binary measures of grasp selection (e.g., Cohen and Rosenbaum, 2004; Herbort and Butz, 2010, 2012; Seegelke et al., 2012, 2013a,b). Furthermore, research on populations with lateralized brain damage can provide intriguing insights into hemispheric specialization in motor planning in the context of object manipulation (e.g., Hermsdörfer et al., 1999; Steenbergen et al., 2000, 2004; Crajé et al., 2009). Interestingly, recent developments in cognitive robotics have opened up new opportunities to examine principles of motor planning in bimanual action. From our point of view, research in motor control can benefit from the advances in technological systems to enhance the understanding of human motor control in skilled unimanual and bimanual voluntary action (e.g., Schack and Ritter, 2013).

## Conflict of Interest Statement

The authors declare that the research was conducted in the absence of any commercial or financial relationships that could be construed as a potential conflict of interest.
